# Updates in Clinical and Translational Glaucoma Research

**DOI:** 10.3390/jcm11010221

**Published:** 2021-12-31

**Authors:** José Javier García-Medina, Maria Dolores Pinazo-Durán

**Affiliations:** 1 Department of Ophthalmology, General University Hospital Morales Meseguer, 30007 Murcia, Spain; 2 Department of Ophthalmology and Optometry, University of Murcia, 30120 Murcia, Spain; 3 Spanish Net of Ophthalmic Research “OFTARED” RD16/0008/0022, Institute of Health Carlos III, 28029 Madrid, Spain; 4 Ophthalmic Research Unit “Santiago Grisolia”/FISABIO, 46017 Valencia, Spain; 5 Cellular and Molecular Ophthalmobiology Group, University of Valencia, 46010 Valencia, Spain; 6 Department of Surgery, Faculty of Medicine and Odontology, University of Valencia, 46010 Valencia, Spain

Glaucoma is a sight-threatening disease and the primum mobile of irreversible blindness worldwide [1]. Throughout the last decade, the interest in glaucoma diagnosis and therapy has been encouraged through outstanding biotechnological advances and the emergence of artificial intelligence to make the decision-making processes of glaucoma management easier [2,3]. Clinicians, biomedical engineers, and scientific researchers have been involved in improving knowledge of glaucoma risk factors and pathogenic mechanisms as well as refining innovative tools for glaucoma diagnostic performance, such as those wielded for corneal biomechanical properties, intraocular pressure (IOP) measurement, structural and functional glaucoma probes [4,5,6,7,8,9], and those regarding the discovery of IOP-lowering medical, laser, and surgical approaches [10,11].

The rising prevalence of diabetes mellitus, hypertensive blood pressure, cardiovascular diseases, respiratory illnesses, neurodegenerative disorders, etc., has had a great impact on global health, and can especially affect the course of glaucoma [12]. The COVID-19 pandemic, causing the severe acute respiratory syndrome coronavirus-2 (SARS-CoV-2), the government/volunteer lockdown, and subjective fear-related restrictions have had a great impact on glaucoma patients [13]. Nevertheless, glaucoma patients may also suffer from ocular disorders that can interfere with vision, quality-of-life, and well-being, such as dry eye disorders, cataracts, uveitis, and/or retinopathies, all of these influencing visual outcomes [14,15].

The primary goal of the Special Issue: “Recent Clinical Research on Glaucoma” is to show readers to review the interdisciplinary background that favors exploration and understand the outstanding preclinical translational research that is taking place in the glaucoma field. The authors of this Special Issue have addressed a series of relevant topics regarding glaucoma risk factors, clinical facts, diagnostic tools, glaucoma comorbidities, treatment, and follow-up, as well as the newest research. A total of 16 works were compiled in this Special Issue, including 1 review, 14 clinical articles and preclinical-translational research studies, and this editorial, which have been collected on this occasion to precisely illustrate the multidisciplinary characteristics of this Special Issue, with the articles synthesized below and ordered by their respective publication dates.

**Sato and Kawaky** [16] take a close look at the “Effects of ripasudil on open-angle glaucoma after circumferential incision of Schlemm’s canal”. Ripasudil hydrochloride hydrate (a Rho-associated coiled-coil containing protein kinase (ROCK) inhibitor), a new type of ocular hypotensive drug, was administered to open-angle glaucoma (OAG) patients who received an operation including a circumferential incision of the Schlemm’s canal and phacoemulsification. The main conclusion was that Ripasudil probably influenced the distal outflow tract by resulting in significant IOP reductions [14].

**Atanasovska Velovska et al.** [17] provided an overview of the “Association of genetic polymorphisms in oxidative stress and inflammation pathways with glaucoma risk and phenotype”. This work fully demonstrated that variability in the interleukin (IL)-1B and -6 gene polymorphisms encode significant risk for glaucoma, while the glutathion peroxidase and tumor necrosis factor gene polymorphism appear to be associated with the glaucoma phenotype.

**Peris Martinez et al.** [18] built upon the current knowledge of the diagnosis and management of glaucoma and keratoconus by using Corvis ST and Pentacam HD devices, through the “Evaluation of intraocular pressure and other biomechanical parameters to distinguish between subclinical keratoconus and healthy corneas”. Therefore, this work demonstrated that the use of normalized biomechanical parameters provided by noncontact tonometry, combined with a discriminant function theory, is a useful tool for detecting subclinical keratoconus in the course of glaucoma.

**Raga-Cervera et al.** [19] analyzed the differential expression profile of miRNAs in the aqueous humor of OHT individuals and glaucoma patients in the analytical, observational, case–control study entitled “miRNAs and genes involved in the interplay between ocular hypertension and primary open-angle glaucoma. Oxidative stress, inflammation and apoptosis networks.” In this work, the authors showed, for the first time, that eight miRNAs expressed differently in tears by comparing OHT and POAG patients. Therefore, Raga-Cervera et al. proposed that specific miRNAs and their target genes and corresponding signaling pathways can be useful to identify HTO individuals at risk of glaucoma neurodegeneration.

**Jeon et al.** [20] showed clinical research results on “Vessel Density Loss of the Deep Peripapillary Area in Glaucoma Suspects and Its Association with Features of the Lamina Cribrosa”. These authors found that glaucoma suspects, with eyes having vessel density defects in the peripapillary area also displayed structural differences in the lamina cribrosa.

Additionally, dealing with the vascular densities of the optic disc areas, **Baek et al.** [21] observed normal-tension glaucoma (NTG) in their work “Optic Disc Vascular Density in Normal-Tension Glaucoma Eyes with or without Branch Retinal Vessel Occlusion”. In conclusion, significant changes in the distribution of vascular densities for the optic disc (in the larger, medium, and small vessels) were observed in both eyes of NTG patients with branch retinal vessel occlusion compared to NTG patients without this condition.

**Del Buey Sayas et al.** [22] analyzed the “Corneal Biomechanical Parameters and Central Corneal Thickness in Glaucoma Patients, Glaucoma Suspects and a Healthy Population” by using an Ocular Response Analyzer (ORA). This work demonstrated that the biomechanical corneal parameters noticeably changed between the above study groups, suggesting that these variables play important roles in glaucoma diagnosis.

**González-Hernández et al.** [23] addressed artificial intelligence tools by means of their innovative article entitled “Fully automated colorimetric analysis of the optic nerve aided by Deep Learning and its association with perimetry for the study of glaucoma”. These authors designed the Laguna-ONhE, an application for the colorimetric analysis of optic nerve images, which is capable of topographically assessing the cup and the presence of hemoglobin. Currently, this tool has been fully improved and automated with five deep-learning models. The authors evaluated glaucoma patients and glaucoma suspects by means of the latest Laguna-OHnE version in combination with perimetry or cirrus-optic coherence tomography, and the results were compared to those from normal eyes. In conclusion, the morphology, perfusion, and function of glaucoma and glaucoma-suspect eyes can be enhanced by using the procedures described herein to provide early sensitivity for better management of glaucoma.

**Chen et al.** [24] performed a nationwide-population-based study on glaucoma risk factors, precisely dealing with “Is Obesity a Risk or Protective Factor for Open-Angle Glaucoma in adults? A Two-Database, Asian, Matched-Cohort Study”. This study aimed to analyze the risk of OAG among obese adults in Taiwan. Data processing of this matched-cohort study at the 13-year follow-up revealed that the obese adults had a higher incidence of OAG and that obese young adults displayed an increased chance of suffering OAG.

A systematic review and meta-analysis were performed by **Liu et al.** [25] on the “Multifocal visual evoked potentials for the detection of visual field defects in glaucoma”. These authors evaluated the diagnostic precision of the mfVEP in glaucoma to find its best diagnostic indicator through the review of quantitative studies published up to 1 April 2021, including a total of 241 patients. The amplitude of mfVEP showed a good diagnostic precision in the prediction of visual field defects. Therefore, Liu et al. suggested that the analysis of the interocular mfVEP amplitude stands as a good indicator for glaucoma diagnosis.

**Gené-Morales et al.** [26] investigated the influence of exercise in IOP by their work entitled “Do age and sex play a role in the intraocular pressure changes after acrobatic gymnastics?” The authors described that the IOP was significantly reduced, and the central corneal thickness remained stable for five minutes after finishing a 90 min acrobatic gymnastics training session. Sex and baseline IOP levels appeared as predictors of IOP variations related to exercise. In summary, acrobatic gymnastics induced IOP reduction, with potentially predicting factors influencing the described changes.

**Fernandez-Albarral et al.** [27] investigated the role of inflammation and immune response and new therapeutic strategies for glaucoma in an animal model. This work was entitled “Is Saffron Able to Prevent the Dysregulation of Retinal Cytokines Induced by Ocular Hypertension in Mice?” The authors utilized a mouse model of unilateral laser-induced OHT, with the main goal of evaluating the production of inflammatory cytokine/chemokine and the changes following saffron treatment. The authors showed that safron extracts had the ability to regulate the expression of pro-inflammatory cytokines, VEGF, and fractalkine, thus protecting the retina from inflammation in the context of OHT.

**Garcia-Medina et al.** [28] carried out the work “Macular structure-function relationships of all retinal layers in primary open-angle glaucoma assessed by microperimetry and 8 × 8 posterior pole analysis of OCT”. The authors fully demonstrated that glaucoma eyes displayed more structure–function relationships than healthy eyes. In addition, it was shown that the associations were positive for the innermost retinal layers but negative for the inner/outer retinal layers in glaucoma eyes. In summary, these data strongly suggest that the inner and outer retinal layers at the macula differ in structure–function relationships.

**Lever et al.** [29] described circulatory changes in early-to-moderate glaucoma in their work entitled “Microvascular and structural alterations of the macula in early to moderate glaucoma: an optical coherence tomography-angiography study”. In this article, the authors reported that glaucoma severity reinforces the relationship between the thickness of the macular segment and the density of vessels. In conclusion, glaucoma individually influences the parameters of the OCT and OCTA.

**Ko et al.** [30], in their observational, cross-sectional study including 1228 eyes from 661 participants (POAG in early, moderate, and later stages, pre-perimetric glaucoma and normal), entitled “Vessel density in the macular and peripapillary areas in preperimetric glaucoma to various stages of primary open-angle glaucoma in Taiwan”, focused on the comparison of the above groups in terms of the changes in peripapillary and macular vessel densities. With this work, the authors stated that the measurements of the peripapillary vessel density could help to distinguish glaucoma stages.

To summarize this collection of papers covering different glaucoma topics, as described above, being the Guest Editors of the Special Issue of the *Journal of Clinical Medicine* **Recent Clinical Research on Glaucoma**, we believe that all of these works can be useful for ophthalmologists, medical specialists, and interdisciplinary researchers to improve our understanding of the pathogenic mechanisms, clinical characteristics, diagnosis, and therapy underlying glaucoma for stimulating innovative “theranostic” glaucoma strategies for better eye and vision care. We sincerely hope that our readers can appreciate the substantial contribution of these works that may contribute to moving this important topic forward.
